# Efficacy and Safety Profile of Novel Oral Anticoagulants in the Treatment of Left Atrial Thrombosis: A Systematic Review and Meta-Analysis

**DOI:** 10.1016/j.curtheres.2022.100670

**Published:** 2022-04-04

**Authors:** Shu-Jie Dong, Cong-Yan Luo, Cui-Lan Xiao, Feng-Zhe Zhang, Lei Li, Zhong-Ling Han, Suo-Di Zhai

**Affiliations:** a Department of Pharmacy, Peking University Third Hospital, Beijing, China; b Department of Pharmacy, Karamay Second People's Hospital, Karamay, China; c School of Pharmaceutical Sciences, Peking University, Beijing, China; d Department of Cardiology and Institute of Vascular Medicine, Peking University Third Hospital, Beijing, China; e Department of Pharmacy, Beijing Road Medical Area of Xinjiang Military Region General Hospital, Urumqi, China

**Keywords:** left atrial, meta-analysis, oral anticoagulants, systematic review, thrombosis, warfarin

## Abstract

**Background:**

The presence of left atrial/left atrial appendage thrombosis is associated with a higher risk of thromboembolic events in patients with atrial fibrillation. The optimal antithrombotic strategy is not established to date.

**Objective:**

Our aim was to compare the efficacy and safety profile of novel oral anticoagulants with warfarin in the treatment of left atrial/left atrial appendage thrombosis.

**Methods:**

We conducted a systematic search in PubMed, Embase, Cochrane Library, ClinicalTrials.gov, and 3 Chinese databases for all randomized controlled trials and cohort studies (PROSPERO, CRD42021238952) from inception to 7 May 2021. Two authors independently performed the articles selection, data extraction, and quality assessment. The efficacy outcome was the resolution of left atrial/left atrial appendage thrombosis, and the safety outcomes were bleeding and stroke/transient ischemic attack.

**Results:**

One randomized controlled trial and 5 cohort studies were included, with a total of 353 patients. Compared with warfarin, novel oral anticoagulants were associated with increased probability of left atrial/left atrial appendage thrombosis resolution (OR = 2.20; 95% CI, 1.35–3.60; *I*^2^ = 0%). Compared with warfarin, novel oral anticoagulants had a similar risk of bleeding (OR = 0.91; 95% CI, 0.39–2.13; *I*^2^ = 0%). There was no evidence of increased risk of stroke/transient ischemic attack (OR = 0.42; 95% CI, 0.12–1.45; *I*^2^ = 0%).

**Conclusions:**

Novel oral anticoagulants were more effective than warfarin in promoting the resolution of left atrial/left atrial appendage thrombosis, without increased risks of bleeding and stroke/transient ischemic attack. Our study provides valuable insight into clinical practice. Further well-designed randomized controlled trials are needed to fully evaluate the benefits and risks in these patients. PROSPERO Registration No.: CRD42021238952.

## Introduction

Atrial fibrillation (AF) is among the most common arrhythmias in clinical practice, with an estimated prevalence of 2% to 4%.^1^ Thromboembolic complications are the leading causes of death and disability in patients with AF. The incidence is 5-fold higher in patients with AF than in patients with normal sinus rhythm. Left atrial (LA) thrombosis, the primary source of thromboembolism in AF, exists in approximately 10% of patients.^2^ Moreover, due to the unique structural characteristics of the left atrial appendage (LAA), 90% of LA thrombosis is located in the LAA in patients with nonvalvular AF (NVAF).^3^ Therefore, anticoagulation therapy is essential to reduce the risk of stroke or other systemic embolic diseases in AF with LA/LAA thrombosis. The optimal antithrombotic strategy of LA/LAA thrombosis resolution has not yet been established.

Currently, the 2020 European Society of Cardiology guidelines^1^ recommend effective oral anticoagulant therapy for at least 3 weeks before reassessment for cardioversion in patients with a thrombosis identified on transoesophageal echocardiography (TEE). Warfarin has been the most-studied anticoagulant agent for treating LA/LAA thrombosis until now. The reported rates of LAA thrombosis resolution range from 16% to 90%.^4^^,^^5^ Unfortunately, the use of warfarin is limited by the narrow therapeutic range, frequent international normalized ratio (INR) monitoring, and dose adjustments.^6^ In addition, the incidence of thromboembolic events remains increased in AF patients with LAA thrombosis while receiving vitamin K antagonists (VKA).^7^

In recent years, novel oral anticoagulants (NOACs) have become attractive alternatives to the long-standing standard of care use of warfarin. NOACs are indicated for prevention and treatment of several cardiovascular conditions. However, the most effective therapeutic use of NOACs in LAA thrombosis need to be established, including the choice of agent and dosing regimens. The purpose of this review was to systematically evaluate the efficacy and safety profile of NOACs for the treatment of LA/LAA thrombosis in patients with NVAF.

## Methods

This systematic review and meta-analysis was performed in accordance with the Preferred Reporting Items for Systematic Review and Meta-analysis statement^8^ and was registered in the International Prospective Register of Systematic Reviews (Registration No.: CRD42021238952).

### Search strategy and study selection

We performed a comprehensive and systematic search in PubMed, Embase, Cochrane Library, Cochrane Central Register of Controlled Trials, ClinicalTrials.gov, and 3 Chinese databases (China National Knowledge Infrastructure, Wanfang, and Chinese Biomedical Literature Database) from inception to May 7, 2021. The systematic search strategy is shown in **Supplemental Table 1**).

Two reviewers (C.Y.L. and F.Z.Z.) performed the selection of the articles independently, and discussed with other authors to reach a consensus when any discrepancies existed between them. The inclusion criteria of the systematic review were in accordance with the PICOS (ie, participants, interventions, comparisons, outcomes and study design) method. Studies that met the following criteria were considered to be included:

Patients: Patients with LA/LAA thrombosis in NVAF detected by TEE;
Intervention: NOACs (including dabigatran, rivaroxaban, apixaban, and edoxaban);
Comparisons: Warfarin;
Efficacy outcomes: The complete resolution of LA/LAA thrombosis;
Safety outcomes: Bleeding, stroke, or transient ischemic attack (TIA); and
Study type: Randomized controlled trials (RCTs) and cohort studies.

Case reports, case series, single-arm studies, meta-analyses, or review articles were excluded. Abstracts only and republished literature were also excluded. No language restriction was imposed. To ensure the publication quality, only Chinese-language articles published in citable Chinese core journals were included.

### Data extraction and quality assessment

#### Data extractions

Two authors (C.Y.L. and F.Z.Z.) extracted data using a collection form independently, and discussed with other authors to reach a consensus when any discrepancies existed between them. We extracted the following data from each study: general information (first author, year of publication, study design); participants (number of eligible subjects, mean age, percentage of men, mean CHA_2_DS_2_-VAS score, mean HAS-BLED score); interventions (type of NOACs, dosage, duration); comparisons (warfarin, INR, duration, time in therapeutic range of INR); outcomes (number of LA/LAA thrombosis resolution, all bleeding events and stroke/TIA).

#### Quality assessment

The quality of the included studies was assessed by 2 authors (C.Y.L. and F.Z.Z.) independently. We assessed the risk of bias of RCTs according to the list of the Cochrane Handbook for Systematic Reviews of Interventions.^9^ We accessed the quality of cohort studies using the Newcastle-Ottawa Scale,^10^ which was based on a semiquantitative principle of the star system, with a total score of 9. The specific contents included population selection, comparability, exposure, or result evaluations. Studies with a score of 7 to 9 were generally indicated as high quality. We compared the quality assessment results, discussed the disagreements, and sought advice from a third reviewer to reach a consensus.

### Data synthesis and analysis

We used the STATA/MP 14.0 statistical software package (StataCorp, Cary, North Carolina) for meta-analysis. Odds ratios (ORs) and 95% CIs of efficacy and safety outcomes were pooled using the Mantel-Haenszel method. Fixed- and random-effect models were fitted according to the heterogeneity estimates. The analysis of heterogeneity among studies was assessed by the *I*^2^ test. When *I*^2^<50%, a fixed-effects model was applied. Otherwise, a random-effect model was more suitable. Because different types of NOACs for LA/LAA thrombolysis were reported in our included studies, we planned to conduct subgroup analysis according to the classification of NOACs (ie, rivaroxaban, dabigatran, apixaban, and edoxaban). A sensitivity analysis was performed to examine the robustness of the pooled results by excluding other types of studies. Finally, a funnel plot analysis was conducted to check for publication bias.

## Results

### Search results

**Figure 1:**
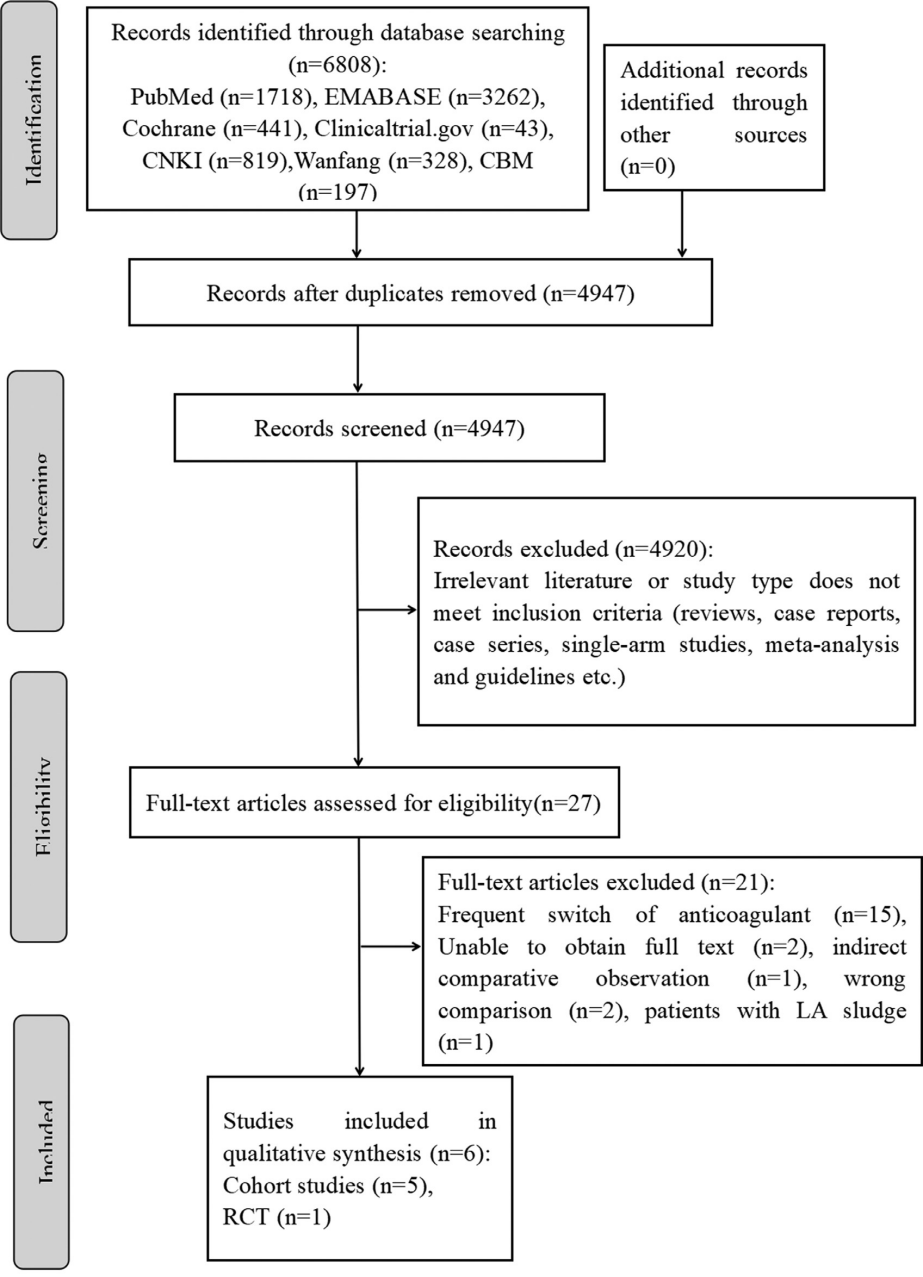
Flow diagram of study selection. CMB = Chinese Biomedical Literature Database; CNKI = Chinese National Knowledge Infrastructure; LA = left atrial; RCT = randomized controlled trial.

Figure 1 shows the results of the literature search and the screening process. A total of 6808 related articles were identified, and 6781 were excluded after the initial screening of titles and abstracts. Full-text screening led to the exclusion of 21 articles for the frequent switch of anticoagulants, indirect comparative observation, wrong comparison, patients with LA sludge, and full text unavailable (Figure 1). Finally, 6 studies^11^, ^12^, ^13^, ^14^, ^15^, ^16^ were included in the systematic review comprising 353 patients in total. Among them, 1^13^ was an RCT and 5^11,12,14–16^ were retrospective cohort studies. Of the 6 studies included, 4^11–14^ were published in English and 2^15,16^ were in Chinese.

### Baseline characteristics and risk of bias

The baseline characteristics of the RCTs and each cohort study are illustrated in Table 1. Of the 6 included studies, 1^13^ specifically compared rivaroxaban and warfarin, 1^11^ compared dabigatran and warfarin alone, and the other 4 had 2 or more kinds of NOACs^12^^,^^14^, ^15^, ^16^ (4 for rivaroxaban, 1 for edoxaban, 2 for apixaban, and 4 for dabigatran). A standard dose was utilized in 5 studies,^11^, ^12^, ^13^, ^14^^,^^16^ and the dose was reduced for patients with renal insufficiency or previous bleeding history. One study^15^ administered rivaroxaban at a dose of 15 mg twice daily for 3 weeks, followed by 20 mg once daily. Two studies described the time in therapeutic range (TTR) of warfarin,^11^^,^^15^ and the other studies only reported the therapeutic range of INR (2.0–3.0). None reported the therapeutic drug monitoring dosage adjustment. Among the included studies, 4 reported the previous use of anticoagulants in patients with LA/LAA thrombosis, and the duration varied from 1 week to 1 month. The follow-up duration ranged from 3 weeks to 1 year. The only RCT in our study had a high risk of bias due to the lack of detailed descriptions of random sequence generation, allocation concealment and blinding implementation (see **Supplemental Figure 1**). The quality assessment of 5 cohort studies is shown in Table 2, and all of them had high quality, with scores >7.

**Table 1:** Baseline characteristics of the included studies.

Study	Hao 2015^11^	Hussain 2019^12^	Ke 2019^13^	Nelles 2021^14^	Lin 2019^15^	Yan 2018^[6^
Country	China	United States	China	Germany	China	China
Study design	Retrospective cohort	Retrospective cohort	RCT	Retrospective cohort	Retrospective cohort	Retrospective cohort
No. of patients	41	45	80	78	14	95
Type of NOAC	Dabigatran, 19 patients	Apixaban, 3 patients Dabigatran, 13 patients Rivaroxaban, 6 patients	Rivaroxaban, 40 patients	Apixaban, 12 patients Dabigatran, 25 patients Rivaroxaban, 11 patients Edoxaban, 1 patients	Dabigatran, 4 patients Rivaroxaban, 6 patients	Dabigatran, 24 patients Rivaroxaban, 40 patients
Dosage of NOAC	150 mg bid	Apixaban 5 mg bid Dabigatran 150 mg bid Rivaroxaban 20 mg qd	20 mg qd	36 (standard dose) 13 (reduced dose)	Dabigatran 110 mg bid Rivaroxaban 15 mg bid for 3 wk, followed by 20 mg qd	Dabigatran 110 mg bid Rivaroxaban 20 mg qd
Therapeutic target of warfarin, INR	2.0–3.0 (TTR >60%)	2.0–3.0	2.0–3.0	2.2–0.2	2.0–3.0 (TTR 75%–100%)	2.0–3.0
Age, y*	57.7 (7.4)	63.2 (15.3)	63.7 (8.6)	76.1 (8.3)	58.92 (10.71)	60.3 (10.6)
Male†	36 (87.8%)	31 (69%)	34 (85%)	45 (57.7%)	13 (93%)	61 (60.4%)
TIA or stroke†	Dabigatran 1 (5%) Warfarin 1(5%)	9 (20%)	1 (2.5%)	23 (29.5%)	NR	19 (18.8%)
CHA2DS2-VASc score*	1.16 (1.01)	3.4 (1.7)	Rivaroxaban, 1.49 (0.82) Warfarin, 1.43 (0.85)	4.3 (1.1)	1.86 (1.75)	NR
HAS-BLED score*	1.21 (1.18)	NR	Rivaroxaban, 1.34 (0.55) Warfarin, 1.42 (0.63)	3.0 (0.7)	1.00 (0.87)	NR
Mean follow-up time	3 mo	67 d	12 wk	116 d	3 mo	≥3 wk
Anticoagulation experienced, %	Dabgatran, 16 Warfarin, 36	<7 d, 100	Untreated within 1 mo, 100	NOAC, 44.9 VKA, 41.0	NR	NR

INR = international normalized ratio; NOACs = novel oral anticoagulants; NR = not recorded; RCT = randomized controlled trial; TIA = transient ischemic attack; TTR = time in therapeutic range; VKA = vitamin K antagonists.

⁎ Values are presented as mean (SD).

† Values are presented as n (%).

**Table 2:** Quality assessment of included cohort studies.

Assessments entry	Hao 2015^11^	Hussian 2019^12^	Nelles 2020^14^	Lin2019^15^	Yan2018^16^
Representativeness of the exposed cohort (1 score)					
Selection of nonexposed cohort (1 score)					
Ascertainment of exposure (1 score)					
Demonstration that outcome of interest was not present at start of study (1 score)					
Comparability of cohorts on the basis of the design or analysis (2 score)					
Assessment of outcome (1 score)					
Was follow-up long enough for outcomes to occur (1 score)					
Adequacy of follow-up of cohorts (1 score)					
Total score (9 scores possible)	8 scores	9 scores	7 scores	8 scores	7 scores

★= score 1 point;  = score 0 point.

### Resolution of LAA thrombosis

**Figure 2:**
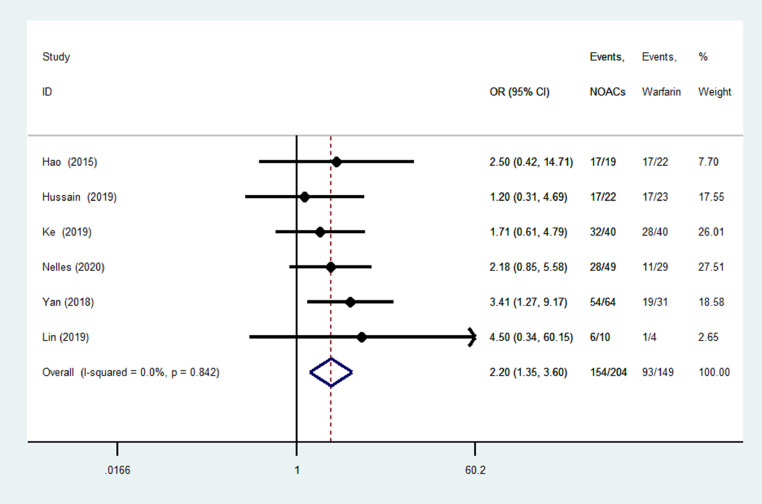
Forest plot of left atrial thrombosis resolution. NOACs = novel oral anticoagulants; OR = odds ratio.

All studies reported the resolution of LA/LAA thrombosis and the definition (the LA/LAA thrombosis had been dissolved, determined by a follow-up TEE) was consistent in the included studies. As shown in Figure 2, we pooled the results of all the included studies. Compared with warfarin (93 out of 149), NOACs (154 out of 204) significantly increased the probability of LA/LAA thrombosis resolution (OR = 2.20; 95% CI, 1.35–3.60; *I*^2^ = 0%). In addition, dabigatran and rivaroxaban significantly increased the resolution of LA/LAA thrombosis compared with warfarin (76.5% vs 59.6%; OR = 2.46; 95% CI, 1.27-4.77; *I*^2^ = 0%; 76.7% vs 59.8%; OR = 2.12; 95% CI, 1.14–3.95; *I*^2^ = 0%) (see **Supplemental Figure 2**). Because the number of patients receiving edoxaban and apixaban was very small, the efficacy profile analysis for these patients was not performed. The funnel plot did not show publication bias (see **Supplemental Figure 3**).

### Bleeding events

**Figure 3:**
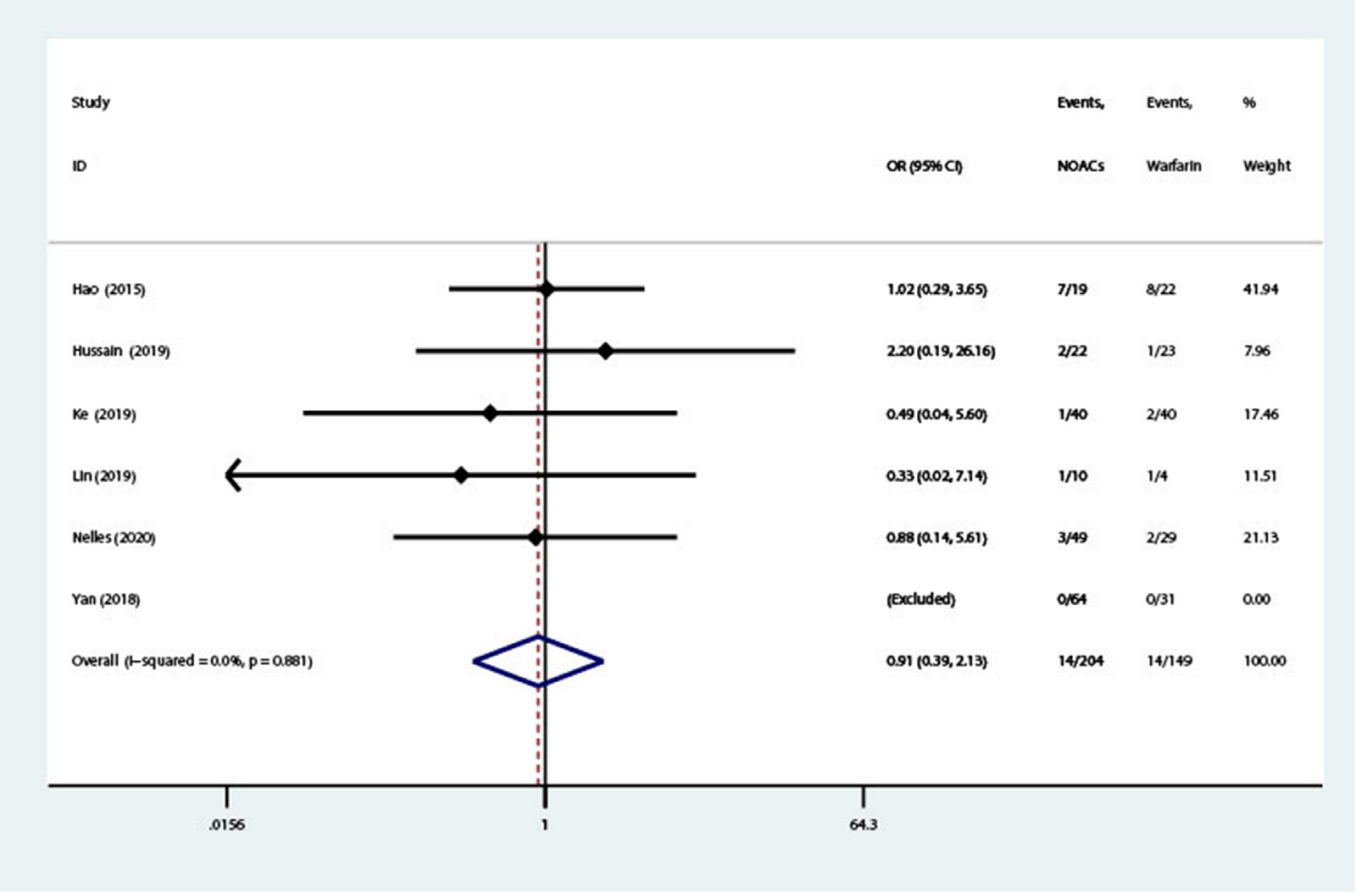
Forest plot of bleeding. NOACs = novel oral anticoagulants; OR = odds ratio.

Bleeding events were reported in 5 cohort studies and 1 RCT (n = 353).^11^, ^12^, ^13^, ^14^, ^15^, ^16^ The definition of bleeding events in 3 studies was in accordance with the International Society on Thrombosis and Haemostasis criteria definition of bleeding events,^11^^,^^14^, ^15^, ^16^ whereas the other 3 studies did not clarify the definition of bleeding events. As shown in Figure 3, NOACs were not associated with increased risk of bleeding compared with warfarin (OR = 0.91; 95% CI, 0.39–2.13; *I*^2^ = 0%). The funnel plot did not show publication bias (see **Supplemental Figure 4**).

### Stroke or TIA

**Figure 4:**
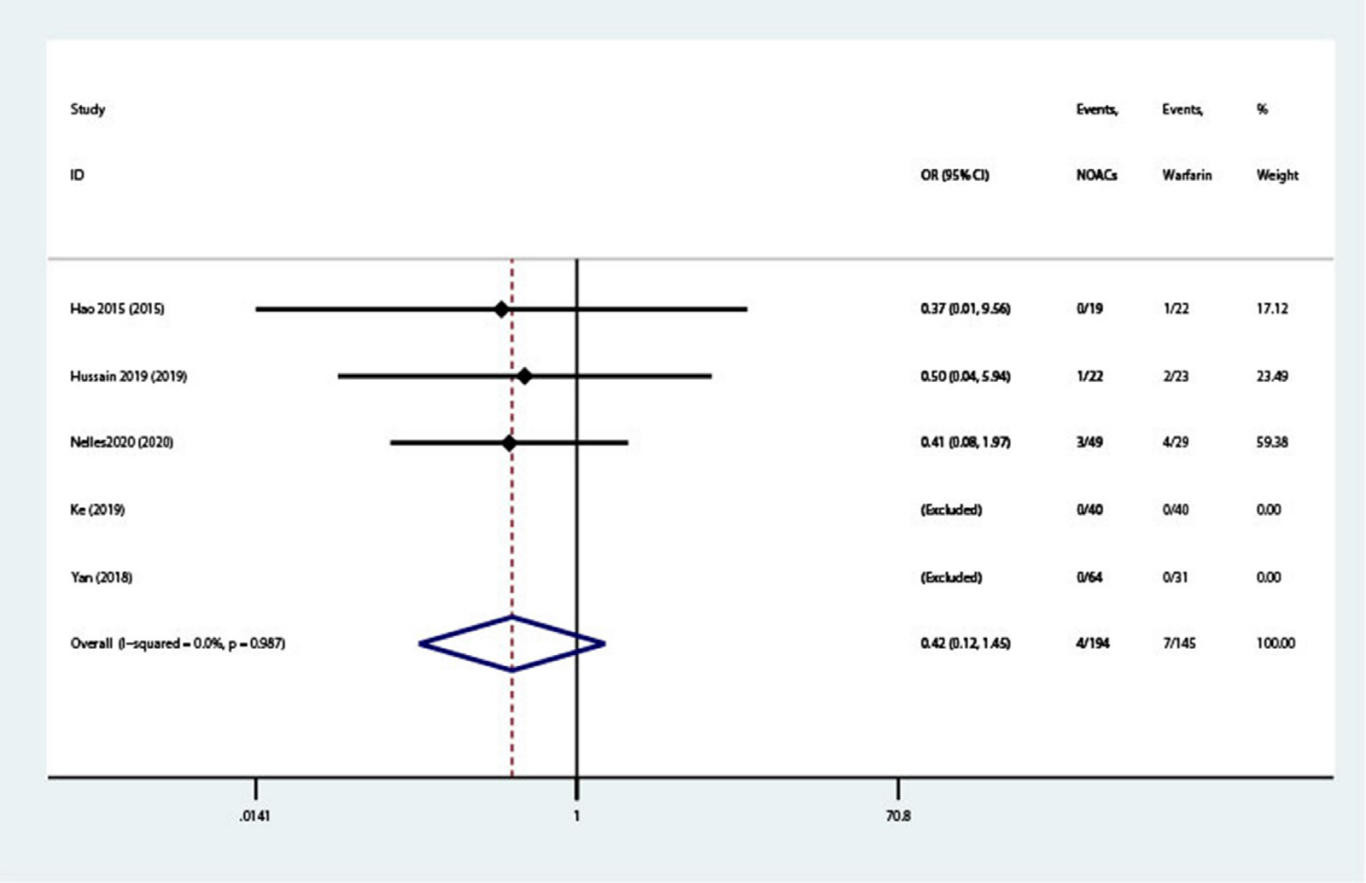
Forest plot of stroke or transient ischemic attach. NOACs = novel oral anticoagulants; OR = odds ratio.

Stroke or TIA were evaluated in 5 studies (n = 339).^11^, ^12^, ^13^, ^14^^,^^16^ As shown in Figure 4, NOACs were not associated with increased risk of stroke or TIA compared with warfarin (OR = 0.42; 95% CI, 0.12–1.45; *I*^2^ = 0%). The funnel plot inspection did not show publication bias (see **Supplemental Figure 5**).

### Sensitivity analysis

We conducted sensitivity analysis by removing the only RCT in our included studies, and the result was consistent with the original pooled size effect (OR = 2.37; 95% CI, 1.36–4.16; *I*^2^ = 0%) (see **Supplemental Figure 6**).

## Discussion

By including 6 studies and 353 participants in total, our study compared the efficacy and safety profile of NOACs with warfarin for treating LA/LAA thrombosis. We found that NOACs were more effective than warfarin in the resolution of LA/LAA thrombosis, without increased risks of bleeding and stroke/TIA. To our knowledge, this is the first study that determined the benefit of NOACs in LA/LAA thrombosis treatment. It has been proven that NOACs are similar or superior to warfarin for the prevention of thromboembolism and stroke.^17^, ^18^, ^19^, ^20^ Moreover, the prevalence of intracardiac thrombi in patients with atrial fibrillation or flutter appears lower with NOACs than with VKA therapy.^21^

A previous meta-analysis^22^ regarding this topic has been published. It concluded that there was no difference in thrombosis resolution between NOACs and warfarin in patients with AF, which was opposite to the results of our study. This previous analysis included a descriptive observation that indirectly compared the results of X-TRA^23^ (a prospective study of participants treated with rivaroxaban alone) and CLOT-AF^24^ (a retrospective registry of participants treated with VKA agents alone). However, the study type, baseline characteristics, and risk of bias were all different between the X-TRA and CLOF-AF studies, which made the comparison between NOACs and warfarin challenging.^25^ This questionable inclusion may have incorrectly altered the direction and magnitude of the pooled effect noted.

The presence of LAA thrombosis is associated with a 2.7-fold higher risk of thromboembolic events.^26^ Thus, the ultimate goal of anticoagulant therapy for LAA thrombosis in patients with NVAF is to prevent thromboembolic events.^27^ Compared with warfarin, NOACs showed a 97% increase in the resolution rate of LA/LAA thrombosis. The pooled resolution rate of the NOACs group in our study was 75.5%, which was also higher than that of VKA (63.7%) in a pooled analysis of 619 patients from 16 VKA studies.^4^ Among different types of NOACs, dabigatran and rivaroxaban both showed a better efficacy profile with respect to the resolution of LA/LAA thrombosis compared with warfarin. The pooled thrombosis resolution rate was 76.5% (65 out of 85) and 76.7% (79 out of 103) for dabigatran and rivaroxaban, respectively. In the included studies, the resolution rate of apixaban was 66.7% (2 out of 3)^12^ and 58.3% (7 out of 12),^14^ respectively, and the resolution rate of edoxaban was 100% (1 out of 1).^14^ Given the limited number of studies and small sample size for each type of NOACs, the rates cannot be compared directly.

With regard to safety profile, the observed occurrence of bleeding events caused by NOACs and warfarin was 7.7% and 11.4%, respectively. However, no significant difference was found between NOACs and warfarin. As reported in a pooled analysis^28^ of RE-LY,^17^ ROCKET-AF,^18^ ARISTOTLE,^19^ and ENGAGE AF-TIMI 48,^20^ a greater relative reduction in bleeding with NOACs was noted at centers that achieved a TTR <66% than at those achieving a TTR ≥66%. Given that maintaining patients’ INRs within the desired range can be difficult for many physicians,^29^^,^^30^ NOACs may be preferred for LAA thrombosis treatment, especially for those who have a poor predicted TTR.

Our findings provide meaningful insights into clinical decision making. Better efficacy and similar safety profile of NOACs make it a more suitable choice in the treatment of LA/LAA thrombosis in patients with NVAF. Because NOACs have been recommended as the first-line therapy for patients with NVAF for years,^1^^,^^31^ their use is strongly preferred in patients with newly detected LA/LAA thrombosis. Clinicians should also assess the concomitant medications, economic conditions, and the compliance of patients individually. The efficacy and safety of warfarin strongly depend on the quality of anticoagulation that is reflected by TTR. Poor TTR control is a major concern in clinical settings and increases the risks of thrombotic and hemorrhagic events.^32^

Currently, the 2020 European Society of Cardiology guidelines^1^ and 2019 American College of Cardiology/American Heart Association guidelines^31^ on AF suggest individual management based on the efficacy (or inefficacy) of previous treatments for LAA thrombosis for at least 3 weeks. As reported, 1.6% to ∼7.7% of patients still had LAA thrombosis detected despite continuous anticoagulant treatment for at least 4 weeks.^33^, ^34^, ^35^, ^36^ According to the RIVA-TWICE study,^37^ for patients who failed to respond to the standard dose, a higher dose of rivaroxaban (15 mg bid) for 8 weeks could further increase the resolution rate of LA/LAA thrombosis to 46.7%. Some studies suggested that prolonged treatment time may contribute to a higher resolution rate, especially for patients with a longer duration of AF.^38^^,^^39^ NOACs also resolved warfarin-resistant thrombosis in some previous reports.^40^^,^^41^ Besides, potentially effective options may also include increasing the target range of INR for warfarin, switching to another type of oral anticoagulants.^42^^,^^43^

There were several limitations in our study. Firstly, the included studies were mostly cohort studies with small sample sizes. Because confounders were not well controlled in some studies,^14^^,^^15^ the reliability of the pooled effect size for unadjusted estimates may be unreliable. Relevant, high-quality RCTs are lacking. Currently, an ongoing RCT (ClinicalTrials.gov ID No.: NCT03792152) is investigating the efficacy of rivaroxaban compared with warfarin in dissolving LAA thrombus in patients with AF that could provide useful information. Secondly, the follow-up time of the included studies varied from 3 weeks to 1 year. Because a longer duration of therapy may lead to improved resolution, the observation and direct comparison of the efficacy and safety outcomes should be interpreted with caution. Thirdly, we were unable to identify whether or not patients in the warfarin groups were maintained at an ideal therapeutic range because only 2 studies reported the TTR of the patients in the warfarin group (TTR >60%). Fourthly, the result of bleeding events would be influenced by inconsistent definitions of bleeding end points in the included studies. Last but not the least, because the sample size is limited, the resolution rate of each type of NOACs cannot be compared directly, and we are unable to further analyze if 1 NOAC is superior to the others.

## Conclusions

NOACs appeared to be more effective than warfarin for the resolution of LA/LAA thrombosis, without the increased risks of bleeding and stroke/TIA. Further well-designed RCTs are needed to fully evaluate the benefits and risks in these patients.

## Appendix Supplementary materials

Image, application 1
