# Supplementary material for: Efficacy and Safety Profile of Novel Oral Anticoagulants in the Treatment of Left Atrial Thrombosis: A Systematic Review and Meta-Analysis
Source: Curr Ther Res Clin Exp. 2022 Apr 4;96:100670. doi: 10.1016/j.curtheres.2022.100670 (PMC9062445; doi:10.1016/j.curtheres.2022.100670)
Supplement: Supplementary file 1 [file mmc1.docx]

**Supplementary material: The search strategy and search results**

PubMed (n=1718), EMBASE (n=3262), Cochrane Library andCENTRAL (n=441)

**PubMed**

#1 "factor xa inhibitors"[MeSH Terms] OR "anticoagulants"[MeSH Terms] OR "rivaroxaban"[MeSH Terms] OR "dabigatran"[MeSH Terms] OR "warfarin"[MeSH Terms] (n=93168)

#2 "direct oral anticoagulants"[Title/Abstract] OR "oral anticoagulants"[Title/Abstract] OR "non vitamin k antagonist"[Title/Abstract] OR "Rivaroxaban"[Title/Abstract] OR "Dabigatran"[Title/Abstract] OR "apixaban"[Title/Abstract] OR "Edoxaban"[Title/Abstract] OR "vitamin k antagonist"[Title/Abstract] OR "antithrombotic agent"[Title/Abstract] OR "factor xa inhibitor"[Title/Abstract] (n=20127)

#3: #1 OR #2 (n=99821)

#4 "thrombosis"[MeSH Terms] (n=132688)

#5 "thrombus"[Title/Abstract] OR "thromboses"[Title/Abstract] OR "blood clot"[Title/Abstract] (n=49310)

#6: #4 OR #5 (n=160256)

#7 "Heart Atria "[MeSH Terms] OR " atrial appendage"[MeSH Terms] (n=43705)

#8 "left atrium"[Title/Abstract] OR "left auricular appendages"[Title/Abstract] OR "Atrial"[Title/Abstract] OR "atrial appendage"[Title/Abstract] OR left atrium appendages (n=175280)

#9: #7 OR #8 (n=187646)

#10: #3 AND #6 AND #9 (n=1718)

**EMBASE**

#1 'warfarin'/exp OR 'blood clotting factor 10a inhibitor'/exp OR 'anticoagulant agent'/exp OR 'apixaban'/exp OR 'antivitamin k'/exp OR 'rivaroxaban'/exp OR 'dabigatran'/exp (n=712028)

#2 warfarin:ab,ti OR 'blood clotting factor 10a inhibitor':ab,ti OR 'anticoagulant agent':ab,ti OR apixaban:ab,ti OR 'antivitamin k':ab,ti OR rivaroxaban:ab,ti OR dabigatran:ab,ti OR 'direct oral anticoagulants':ab,ti OR 'oral anticoagulants':ab,ti OR 'non vitamin k antagonist':ab,ti OR 'factor xa inhibitor':ab,ti OR 'antithrombotic agent':ab,ti (n=64921)

#3 'thrombus'/exp OR 'blood clot'/exp OR 'thrombosis'/exp (n=362717)

#4 thrombus:ab,ti OR 'blood clot':ab,ti OR thrombosis:ab,ti (n=258592)

#5 'heart atrium'/exp OR 'heart atrium appendage'/exp (n=88865)

#6 'heart atrium':ab,ti OR 'heart atrium appendage':ab,ti OR 'left atrium':ab,ti OR 'left auricular appendages':ab,ti OR 'left atrium appendages':ab,ti (n=25523)

#7: #1 OR #2 (n=715024)

#8: #3 OR #4 (n=427797)

#9: #5 OR #6 (n=97438)

#10: #7 AND #8 AND #9 (n=3262)

**Cochrane Library,CENTRAL**

#1 MeSH descriptor: [Heart Atria] explode all trees (n=586)

#2 (left atrium):ti,ab,kw OR (left auricular appendages):ti,ab,kw OR (atrial):ti,ab,kw OR (atrial appendage):ti,ab,kw OR (left atrium appendage):ti,ab,kw" (Word variations have been searched) (n=18188)

#3 MeSH descriptor: [Thrombosis] explode all trees (n=4894)

#4 (thromboses):ti,ab,kw OR (thrombus):ti,ab,kw OR (blood clot):ti,ab,kw AND (blood clots):ti,ab,kw AND (clot, blood):ti,ab,kw (Word variations have been searched)" (Word variations have been searched) (n=10169)

#5 MeSH descriptor: [Factor Xa Inhibitors] explode all trees (n=568)

#6 (direct oral anticoagulants):ti,ab,kw OR (oral anticoagulants):ti,ab,kw OR (non vitamin k antagonist):ti,ab,kw OR (Rivaroxaban):ti,ab,kw OR (Dabigatran):ti,ab,kw" (Word variations have been searched) :ti,ab,kw OR (apixaban):ti,ab,kw OR (Edoxaban):ti,ab,kw OR (vitamin k antagonist):ti,ab,kw OR (antithrombotic agent):ti,ab,kw OR (factor xa inhibitor):ti,ab,kw" (Word variations have been searched) (n=475355)

#7：(#1 OR #2)AND (#3 OR #4) AND (#5 OR #6) (n=441)

**Supplementary material:Figure**


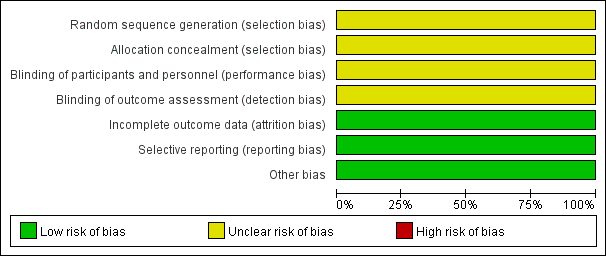


Figure S1. Risk of bias for one RCT in this study


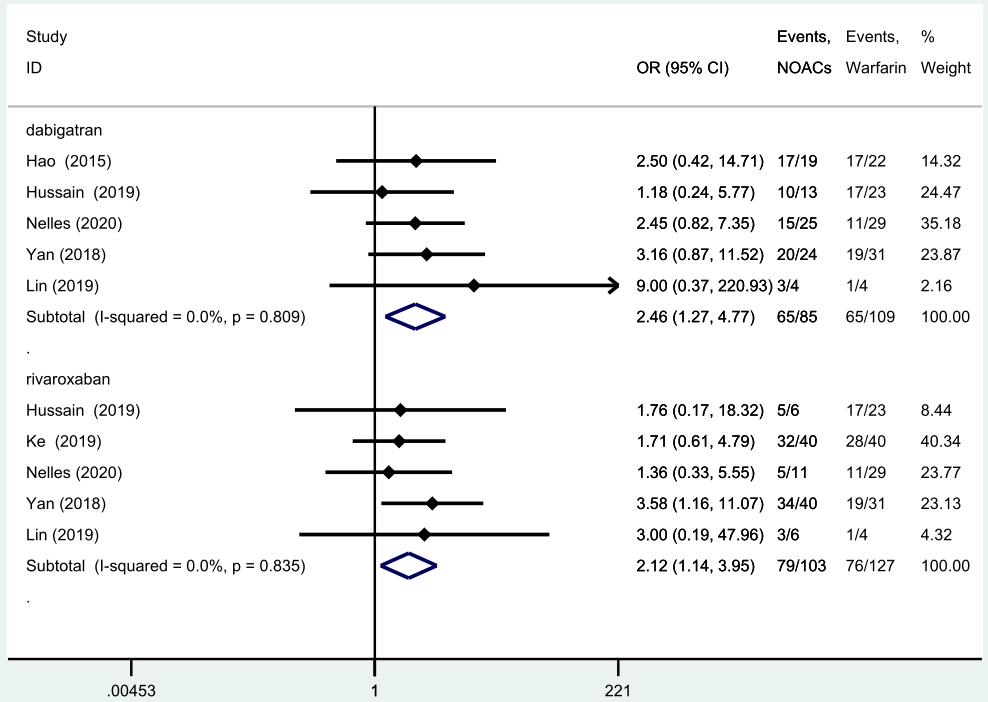


Figure S2. Subgroup analysis


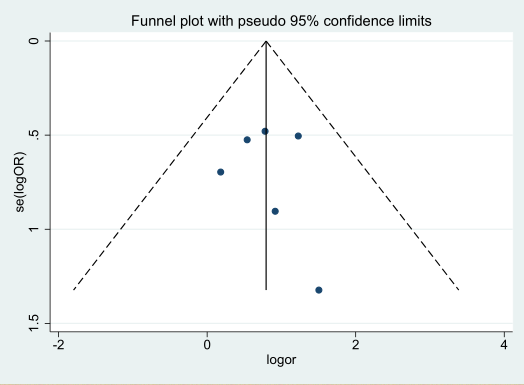


Figure S3. Funnel plot of comparison for LA/LAA thrombosis resolution


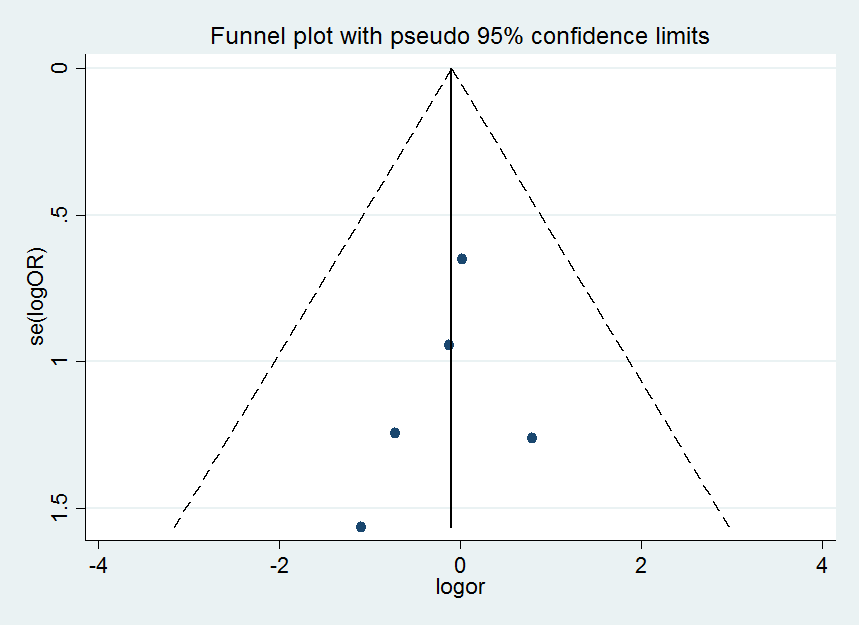


Figure S4. Funnel plot of comparison for bleeding events


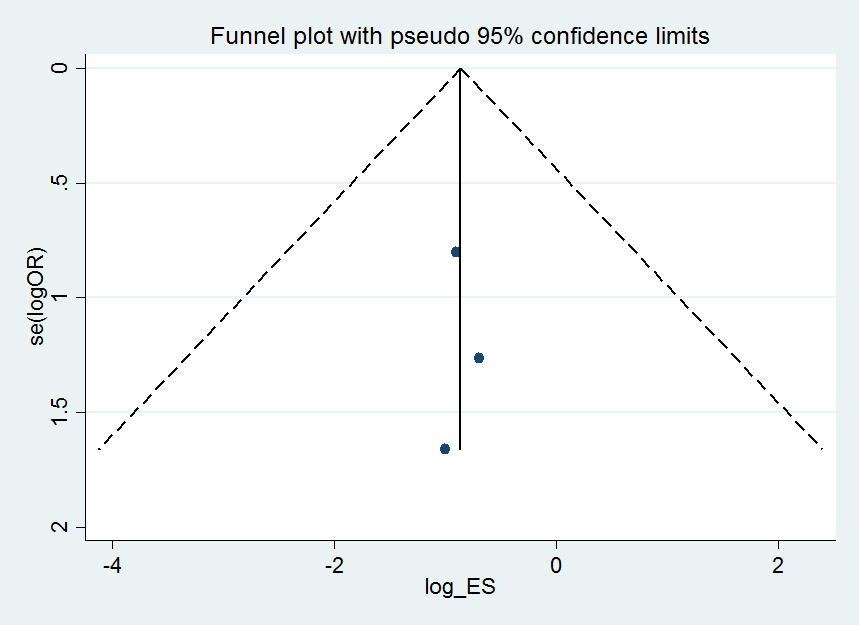


Figure S5. Funnel plot of comparison for stroke/TIA


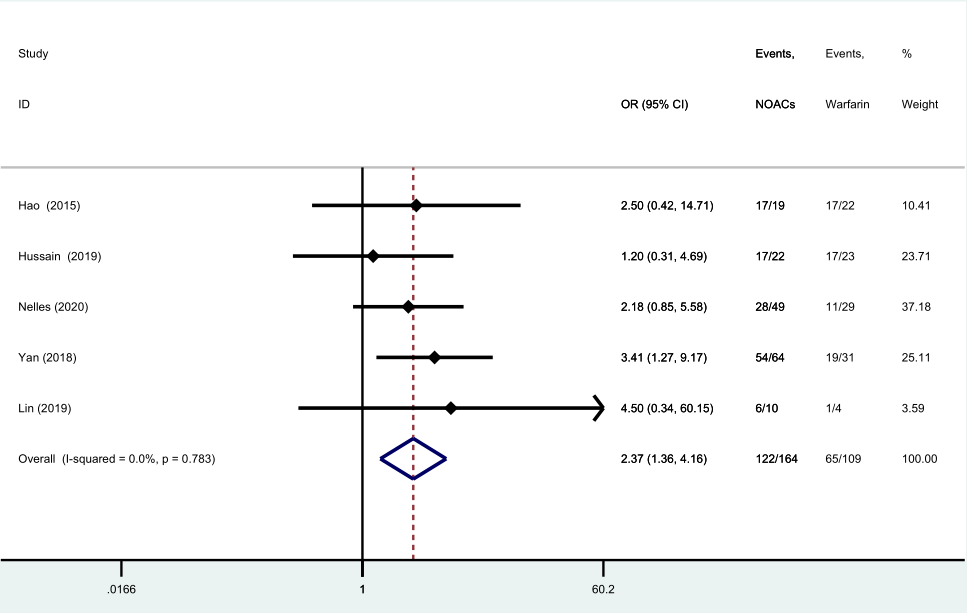


Figure S6. Sensitivity analysis by removing the only RCT
